# Features of the Atrophic Corpus Mucosa in Three Cases of Autoimmune Gastritis Revealed by Magnifying Endoscopy

**DOI:** 10.1155/2012/368160

**Published:** 2012-07-03

**Authors:** Kazuyoshi Yagi, Atsuo Nakamura, Atsuo Sekine, David Graham

**Affiliations:** ^1^Department of Internal Medicine, Niigata Prefectural Yoshida Hospital, Tsubame, Niigata 959-0242, Japan; ^2^Department of Medicine, Michael DeBakey Veterans Affairs Medical Center and Baylor College of Medicine, Houston, TX 77030, USA

## Abstract

Atrophic gastritis, whether caused by *Helicobacter pylori* infection or as a result of an autoimmune process, is associated with corpus atrophy. However, whereas atrophic gastritis caused by *H. pylori* involves the antrum, the antrum is spared in autoimmune gastritis. Here, we report the use of magnifying endoscopy to identify and distinguish atrophic gastritis caused by *H. pylori* from autoimmune gastritis. The mucosal pattern in autoimmune gastritis is that of closely arranged small round and oval pits, thus differing from the pattern seen in atrophic mucosa due to *H. pylori* infection. We speculate that this reflects differences in inflammation between the two types of gastritis. In autoimmune gastritis the inflammation is directed primarily against gastric glands, whereas in *H. pylori* infection the inflammation is directed against the bacteria on or near the surface and the damage initially affects the surface epithelium. During repair, the normal regular round pits are destroyed, whereas they remain largely intact in mucosa with autoimmune-associated atrophy. Confirmation of the features of autoimmune gastritis revealed by magnifying endoscopy would not only make the endoscopic diagnosis of autoimmune gastritis more accurate, but also help to elucidate changes in the surface epithelial structure of gastritis due to various causes.

## 1. Introduction

The two most common causes of atrophic gastritis are an autoimmune mechanism or persistent infection with *Helicobacter pylori*. Ultimately, persistent chronic inflammation may result in severe atrophy of the stomach's mucosal lining. In autoimmune gastritis the antral mucosa is spared, and characteristically the atrophy is associated with a marked elevation of the serum gastrin level, which may result in type I gastric carcinoid tumors [1, 2]. In contrast, *H. pylori* infection affects the gastric antrum and tends to blunt the hypergastrinemic response to hypochlorhydria, gastric carcinoids being uncommon. Although both of these two forms of atrophic gastritis are common, there have been no reports of endoscopically evident features that can reliably distinguish between autoimmune and *H. pylori* induced atrophic gastritis.

Here we used magnifying endoscopy to identify differences between the two types of atrophic gastritis. The appearance of the gastric corpus in patients with autoimmune gastritis differs from that of atrophic gastritis caused by *H. pylori* infection, which exhibits either ridged surface structures or a villous to granular surface pattern [3–6]. In contrast, the magnified endoscopic appearance of the atrophic mucosa in autoimmune gastritis is similar, but not identical, to that of the corpus in the *H. pylori*-negative stomach. We report our findings obtained using magnifying endoscopy in three patients with autoimmune gastritis. We found that the atrophic mucosa of the corpus in all three patients exhibited a previously undescribed pattern of closely arranged small round and oval pits.

## 2. Case Reports

### 2.1. Case 1

 A 48-year-old woman presented with epigastric discomfort. Upper gastrointestinal (GI) endoscopy revealed severe mucosal atrophy in the gastric corpus. Magnifying endoscopy of the corpus revealed closely arranged small round and oval pits with a surrounding network of capillaries. In the antrum, both conventional and magnifying endoscopy demonstrated no abnormal features, and the patient was tentatively diagnosed as having autoimmune gastritis [3–6]. Biopsy specimens were taken from both the antrum and the corpus. The histology of the antral mucosa was normal, whereas that of the corpus exhibited severe atrophy. The serum gastrin level was 4440 pg/mL (reference range: <200 pg/mL), and the level of antiparietal cell antibody was ×160 positive. The patient was negative for anti-intrinsic factor antibody and anti-*H*.  *pylori* antibody IgG, and the urea breast test gave a result of 2.2‰  (reference range: <2.5‰). The diagnosis of autoimmune gastritis was therefore confirmed.

### 2.2. Case 2

**Figure 1:**
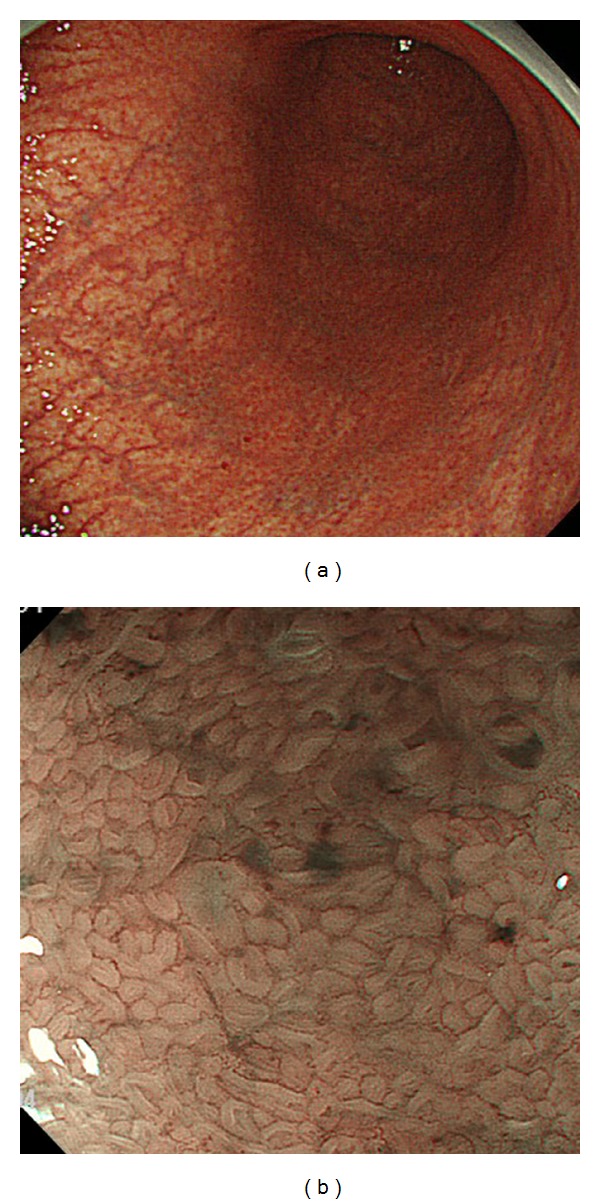
(a) Conventional endoscopic finding in the corpus of Case 2. (b) Magnifying endoscopic findings in the corpus of Case 2 with narrow band imaging (NBI) (full zoom).

 A 72-year-old woman presented for a routine health check. Upper GI endoscopy identified severe atrophy of the mucosa in the gastric corpus (Figure 1(a)), and magnifying endoscopy showed a pattern of closely spaced small round and oval pits (Figure 1(b)). Conventional endoscopy of the antrum demonstrated no abnormality, suggesting a diagnosis of autoimmune gastritis. As this endoscopic examination was part of a routine health check, no biopsy was performed. However, serum samples revealed a gastrin level of 1800 pg/mL (reference range: <200 pg/mL) and antiparietal cell antibody ×40 positive, with negativity for anti-intrinsic factor antibody and anti-*H. pylori* antibody IgG. Autoimmune gastritis was therefore confirmed.

### 2.3. Case 3

**Figure 2:**
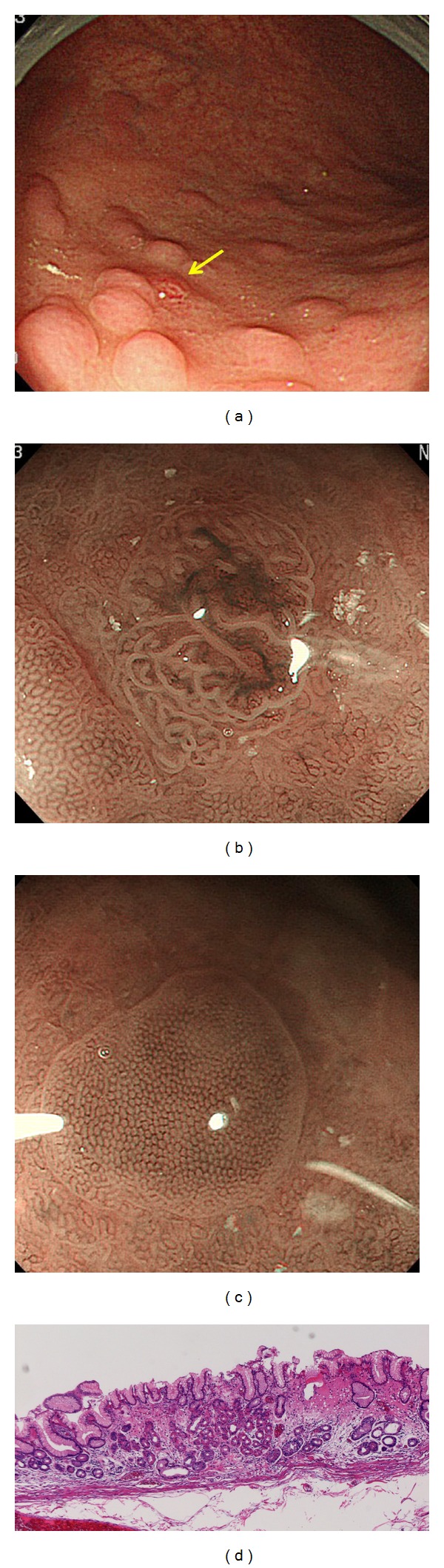
(a) Conventional endoscopic findings in Case 3. Yellow arrow shows a nodule with redness. (b) Magnifying endoscopic appearance of the nodule with redness in Figure 2(a) (full zoom). (c) Magnifying endoscopic appearance of the other nodules in Figure 2(a) (full zoom). (d) Histological appearance of the other nodules in Figure 2(c). Atrophic mucosa with fundic gland remnants is evident.

 A 58-year-old man had been found to have gastric nodules by X-ray and endoscopic examinations at another hospital. During the endoscopic examination, three nodules were biopsied and one was suspected to be a carcinoid tumor. The other two were diagnosed as atrophic gastric mucosa. A conventional endoscopic reexamination at our hospital revealed multiple nodules on the greater curvature of the corpus (Figure 2(a)), one of which was reddish (Figure 2(a) yellow arrow). Magnifying endoscopy showed that this reddish nodule had a gyriform-like structure with an irregular vascular pattern (Figure 2(b)). Based on the magnifying endoscopy findings, the reddish nodule was diagnosed as a carcinoid tumor, and this was subsequently confirmed by histological examination of a biopsy sample. Conventional endoscopic examination of the corpus demonstrated severe atrophic gastritis (Figure 2(a)). Magnifying endoscopic examination of the other nodules revealed closely arranged small round pits (Figure 2(c)) and a pattern of closely arranged small round and oval pits was also observed in the mucosa surrounding these nodules (Figures 2(b) and 2(c)). Thus, magnifying endoscopy was able to show that this characteristic pit pattern was present even in the atrophic mucosa of the corpus, leading to a diagnosis of autoimmune gastritis associated with a carcinoid tumor. Analysis of the patient's serum showed a gastrin level of 2700 pg/mL (reference range: <200 pg/mL), antiparietal cell antibody ×80 positive, positivity for anti-intrinsic factor antibody, and negativity for anti-*H. pylori* antibody IgG. The reddish nodule and some of the surrounding nodules that showed closely arranged small round pits were treated by endoscopic mucosal dissection (ESD). The reddish nodule was 3 mm in diameter and histological examination confirmed that it was a carcinoid tumor. The other small nodules showed remnants of fundic glands within the atrophic mucosa (Figure 2(d)).

## 3. Discussion

Strickland and Mackay described autoimmune gastritis as Type A atrophic gastritis [1]. One characteristic of autoimmune gastritis is hypergastrinemia, which may lead to the development of gastric carcinoids [1], the histological features of autoimmune gastritis are a normal pyloric glandular mucosa and severe atrophic changes in the corpus, thus differing from those of atrophic gastritis caused by *H. pylori* infection, which primarily affects the antrum. The endoscopic diagnosis is based on an apparently normal antral mucosa alongside severe atrophic mucosa in the corpus. However, it difficult to reliably diagnose autoimmune gastritis on the basis of this criterion alone. Histologically, normal pyloric gland mucosa is taken from antrum, although atrophic mucosa is taken from the corpus in biopsy specimens. However, in a real clinical condition, a diagnosis is only rarely decided from biopsy specimens, and instead, is based on the results of serological examinations for gastrin, antiparietal cell antibody and anti-intrinsic factor antibody. A much more practical approach would be to diagnose autoimmune gastritis by imaging of the morphological features, that is, by using magnifying endoscopy, before serological confirmation.

We have previously described the characteristic features of the corpus in *H. pylori* infection revealed by magnifying endoscopy [3–5] and classified such *H. pylori* induced gastritis into four types (the so-called “Z classification”) which excludes an atrophic mucosa [4, 5]. However, in order to include cases showing an atrophic mucosa, the classification was subsequently modified and renamed the A-B classification system [3, 6]. The normal mucosa of the corpus without *H. pylori* infection, as demonstrated by magnifying endoscopy, shows a pattern of regular round pits surrounded by a network of capillaries that coalesce into collecting venules; this is classified as type B-0 [3–6]. Mild gastritis due to *H. pylori* shows round pits surrounded by a slightly irregular network of capillaries, and this is classified as type B-1 [3–6]. Active gastritis showing round pits with dividing sulci is classified as type B-2, and active gastritis with dilated pits and denser sulci is classified as type B-3 [3–6]. Atrophic mucosa with ridge surface structures is classified as type A-1 and that with villous to granular surface structures as type A-2 [3, 6].

Magnifying endoscopy shows that atrophic mucosa has an appearance different from that of nonatrophic mucosa. In case 1, closely arranged small round and oval pits were seen in the atrophic mucosa and, based on the above classifications, we considered that the patient had autoimmune gastritis. From this experience, we were able to diagnose cases 2 and 3 as autoimmune gastritis based on these magnifying endoscopic features, which differ from those of the atrophic mucosa in *H. pylori* infection and are similar but not identical to those of the corpus in the absence of *H. pylori* (type B-0).

We think that this feature of autoimmune gastritis revealed by magnifying endoscopy is very practical for diagnostic confirmation before ordering serological examination.

Additional cases of autoimmune gastritis studied by magnifying endoscopy are needed to confirm that the pattern described above is truly a unique feature of the corpus in this disease. It is interesting to note that the magnified view of the atrophic mucosa in the corpus of patients with autoimmune gastritis differs from that seen in *H. pylori* induced mucosal atrophy. We speculate that this may be related to the differences in the pattern and type of inflammation between the two types of gastritis. In autoimmune gastritis, the inflammation is largely mononuclear and directed primarily against gastric glands, whereas in *H. pylori* infection the inflammation shows an acute on chronic pattern and is directed against the bacteria located on the surface. In *H. pylori* gastritis, the mucosal damage is largely a bystander effect, and most severe at the surface epithelium. Ongoing inflammation likely influences mucosal repair and does not allow reconstruction of the normal surface epithelium (i.e., the regular round pits are destroyed). In contrast, in autoimmune gastritis, the normal surface structure may remain intact or be subjected to less damage, remaining recognizable in the atrophic mucosa as a pattern of closely arranged round and oval pits.

The changes of surface epithelial structure in various types of gastritis are thought to differ according to cause, and the features revealed by magnifying endoscopy may provide clues for elucidating such changes in surface epithelial structure.

Furthermore, a gyriform-like structure surrounded by closely arranged small round pits in the atrophic mucosa, as seen in Case 3, may be a specific magnifying endoscopic feature of carcinoid arising from autoimmune gastritis. Further accumulation of cases of autoimmune gastritis, observed by magnifying endoscopy, will be necessary.

In conclusion, magnifying endoscopy has shown that, in autoimmune gastritis, the atrophic mucosa in the corpus has a characteristic pattern of closely arranged small and oval pits, thus differing markedly from the appearance of atrophic gastritis caused by *H. pylori* infection. Magnifying endoscopic observation is thus very useful for diagnosis of autoimmune gastritis. Confirmation of the magnifying endoscopic features of autoimmune gastritis would not only make the endoscopic diagnosis of autoimmune gastritis more accurate but also help to clarify the changes in surface epithelial structure characteristic of gastritis due to various causes.
